# Insights into treatment-specific prognostic somatic mutations in NSCLC from the AACR NSCLC GENIE BPC cohort analysis

**DOI:** 10.1186/s12890-024-03124-4

**Published:** 2024-07-02

**Authors:** Yi Liu, Sindhu Yalavarthi, Fan Yang, Yusif Abdul-Rashid, Shenkun Tang, Zihe Long, Yongkai Qin, Kerui Wu, Zhifei Wang

**Affiliations:** 1 Department of Neurosurgery, the Third XiangYa Hospital of Central South University, Changsha, 410013 PR China; 2 Department of Nanoscience, The Joint School of Nanoscience and Nanoengineering, University of North Carolina Greensboro, Greensboro, NC 27401 USA

**Keywords:** Clinocogenomic, NSCLC, Treatment response, Mutational signature, GENIE

## Abstract

**Background:**

Treatment of non-small lung cancer (NSCLC) has evolved in recent years, benefiting from advances in immunotherapy and targeted therapy. However, limited biomarkers exist to assist clinicians and patients in selecting the most effective, personalized treatment strategies. Targeted next-generation sequencing–based genomic profiling has become routine in cancer treatment and generated crucial clinicogenomic data over the last decade. This has made the development of mutational biomarkers for drug response possible.

**Methods:**

To investigate the association between a patient’s responses to a specific somatic mutation treatment, we analyzed the NSCLC GENIE BPC cohort, which includes 2,004 tumor samples from 1,846 patients.

**Results:**

We identified somatic mutation signatures associated with response to immunotherapy and chemotherapy, including carboplatin-, cisplatin-, pemetrexed- or docetaxel-based chemotherapy. The prediction power of the chemotherapy-associated signature was significantly affected by epidermal growth factor receptor (*EGFR*) mutation status. Therefore, we developed an *EGFR* wild-type–specific mutation signature for chemotherapy selection.

**Conclusion:**

Our treatment-specific gene signatures will assist clinicians and patients in selecting from multiple treatment options.

**Supplementary Information:**

The online version contains supplementary material available at 10.1186/s12890-024-03124-4.

## Backgroud

Lung cancer is the leading global cause of death, accounting for an estimated 2 million diagnoses and 1.8 million deaths annually [1]. Based on cell origin, lung cancer comprises two main types: small cell lung cancer (SCLC) and non-small cell lung cancer (NSCLC) [2]. Substantial improvements in lung cancer treatment, particularly in NSCLC, have emerged over the past decades [3]. The transformative progress in this field, influencing treatment preferences and allowing for individualized sequencing strategies, has ushered in the precision treatment era [4–6]. While chemotherapy remains a prevalent first-line therapy, there has been a noticeable shift in patient preference towards immunotherapy, chemo-immunotherapy, and targeted therapy [4–6]. The evolving clinical landscape necessitates the development of methods aiding clinicians and patients in selecting personalized and effective treatment strategies.

Recently, numerous studies have demonstrated mutational analyses within clinical cohorts across various settings, including therapy responses. For instance, Smith et al. demonstrated that patients with different mutations respond differentially to chemotherapy and immunotherapy [7]. Shire et al. reported that STK11 negatively affects lung cancer patients' survival [8]. Genomic profiling has gained increasing importance in cancer diagnosis and treatment. With the decreasing cost and the rise of approved commercial next-generation sequencing platforms, genomic profiling has become routine in many cancer types, and millions of tumors have been sequenced over the past decade.

To facilitate the sharing of ‘big data’ across different institutes, the American Association for Cancer Research (AACR) launched the Genomics Evidence Neoplasia Information Exchange (GENIE) in 2016 as a publicly accessible registry of real-world clinical and genomic data from cancer patients [9]. In collaboration with 10 biopharmaceutical companies, AACR initiated the Biopharma Collaborative (BPC) in 2019, aiming to add deep clinical annotation to select cohorts of patients from the main GENIE Registry [10]. In 2023, the NSCLC GENIE BPC cohort was released as one of the six cohorts featuring detailed clinical and genomic curation of 2,004 tumor samples from 1,846 patients [10]. The GENIE BPC cohort includes meticulously curated data on treatment and progression for each sequenced patient. Therefore, through an in-depth exploration of this invaluable dataset, we sought to gain insights into mutations associated with treatment responses.

In clinical practice, lung cancer patients predominantly receive immune therapy, targeted therapy, and chemotherapy. Targeted therapy, strictly dictated by genetic alterations like the EGFR mutations, is not a substitute but a complementary aspect to immunotherapy or chemotherapy. Our study aimed to identify prognostic markers for immunotherapy and chemotherapy, which is crucial for informed decision-making in the presence of multiple treatment options.

## Materials and methods

### Data acquisition and processing

Clinical data (GENIE BPC NSCLC v2.0-public) and genomic data (Release 13.0-public) were obtained from cBioPortal and Sage Bionetworks, respectively. Patients in the GENIE BPC cohort underwent sequencing using 11 different gene panels (sTable 1). Three of these panels were excluded due to the limited number of sequenced genes. We focused our analysis on the 158 shared genes across the remaining eight panels (sTable 2). Progression and regimen information were curated according to the guidelines provided by the GENIE BPC team [10]. PFS based on imaging from the clinical data was utilized for subsequent analyses. Samples with unavailable sequencing results were excluded from the analysis. For patients with multiple sequenced samples, only one sample was retained for analysis. Samples were excluded based on the following criteria: 1) in cases where multiple samples exhibited identical sequencing results (majority), one sample was randomly selected and retained for analysis; 2) in instances involving multiple samples with different sequencing results, the earliest sample that underwent sequencing was retained for analysis. For histology subtype, patients were analyzed based on Oncotree code [11]. Besides LUAD and LUSC, minor histology subtype including LCLC, LUAS, SARCL, LUNE, NSCLCPD, LUPC, NSCLC were grouped as others.

### Statistical analysis

Multivariate analysis was conducted using the R survival package (https://github.com/therneau/survival), while Cox proportional hazard regression was calculated with the R glmnet package (https://glmnet.stanford.edu). Survival analyses, employing the Kaplan–Meier method, were conducted using GraphPad Prism version 9.0 (https://www.graphpad.com/updates/prism-900-release-notes), and the significance of differences was assessed through log-rank tests. Chi-square tests were performed using the R stats package (https://stat.ethz.ch/R-manual/R-devel/library/stats/html/00Index.html). Co-occurrence analysis utilized the Fisher exact test, implemented with the R maftools package [12].

### Genomic visualization

The mutational co-occurrence and oncoplots were generated using the R maftools package [12].

## Results

### Clinical characteristics and treatment outcomes in NSCLC patients

**Fig. 1:**
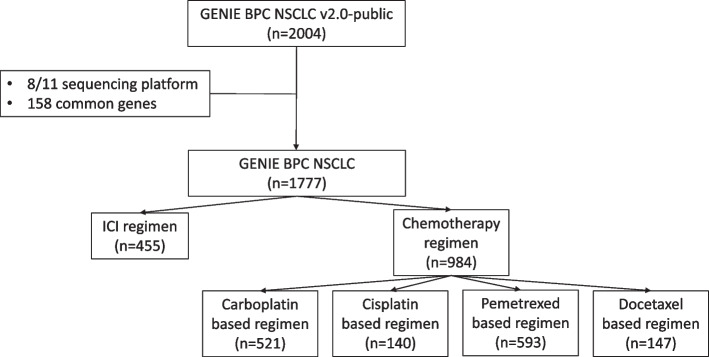
Data acquisition and processing

To investigate the association of somatic mutation with patient’s response to specific treatment, we employed the NSCLC GENIE BPC cohort, consisting of 2,004 tumor samples from 1,846 patients sequenced by 11 different gene panels (sTable 1). The clinical data (GENIE BPC NSCLC v2.0-public) and genomic data (Release 13.0-public) were obtained from cBioPortal and Sage Bionetworks, respectively. By combining the gene panels, we identified 158 common genes sequenced from a total of 1,777 tumors from 1,637 patients (Fig. 1). Notably, the majority of these patients underwent multiple treatments, with an average of 2.14 cancer-directed drug regimens curated for each patient.

Within this dataset, we identified 455 immune checkpoint inhibitor (ICI) regimens and 984 chemotherapy regimens. For ICI regimens, we conducted a multivariate Cox regression analysis to explore the association of age, race, sex, stage, smoking status, PDL1 status, and prescribed drug with the progression-free survival index (Table 1). Consistent with previous research [13–15], PDL1 status and smoking status were found to be significantly related to progression-free survival (PFS) in patients treated with ICI regimens.

**Table 1:** Characteristic of patients treated with ICI

	*N* = 455	Hazard Ratio	*p* value
**Age**			
Median [Min, Max]	65.56[30.2,87.5]	0.99(0.98–1.00)	0.23
**Race**			
AAPI	31 (6.8%)	Reference	
Black	27 (5.9%)	1.28(0.70–2.33)	0.41
White	370 (81.3%)	1.16(0.75–1.80)	0.48
Other	12 (2.6%)	0.62(0.26–1.46)	0.28
Unknown	15 (3.3%)	0.73(0.35–1.49)	0.39
**Sex**			
Female	228 (50.1%)	Reference	
Male	227 (49.9%)	0.97(0.79–1.20)	0.83
**Stage**			
I	38 (8.4%)	Reference	
II	42 (9.2%)	0.82(0.48–1.40)	0.48
III	83 (18.2%)	1.08(0.69–1.69)	0.73
IV	291 (64.0%)	1.24(0.84–1.85)	0.27
**Smoking statues**			
Current	75 (16.5%)	Reference	
Former	304 (66.8%)	1.22(0.91–1.63)	0.18
Never	76 (16.7%)	1.56(1.07–2.28)	0.02
**PD-L1 statues**			
Negative	68 (14.9%)	Reference	
Positive	135 (29.7%)	0.74(0.54–1.00)	0.05
NA	252 (55.4%)	0.61(0.41–0.91)	0.02
**Histology**			
LUAD	360 (79.1%)	Reference	
LUSC	59 (13.0%)	1.03(0.75–1.40)	0.85
Other	36 (7.9%)	0.69(0.46–1.03)	0.07
**ICI**			
Atezolizumab	50 (11.0%)	Reference	
Nivolumab	237 (52.1%)	0.96(0.68–1.35)	0.83
Pembrolizumab	168 (36.9%)	1.03(0.70–1.51)	0.86

Additionally, a multivariate Cox regression analysis was performed to investigate the association of age, race, sex, stage, smoking status, and prescribed regimen with PFS for chemotherapy (Table 2). Age and sex demonstrated significant associations with PFS in chemotherapy, aligning with earlier studies [16, 17]. The analysis of regimen-specific PFS revealed a significant difference among the groups. As reported previously, cisplatin- and platinum-based chemotherapies showed longer PFS compared to carboplatin-based therapy [18]. In contrast, platinum-based therapy, in general, exhibited longer PFS compared to non-platinum-based therapy [19].

**Table 2:** Characteristic of patients treated with chemotherapy

	*N* = 984	Hazard Ratio	*p* value
**Age**			
Median [Min, Max]	64.0 [30.1, 87.5]	0.99(0.98–0.99)	0.0386
**Race**			
AAPI	85 (8.6%)	Reference	
Black	58 (5.9%)	0.89(0.58–1.35)	0.5947
White	781 (79.4%)	0.82(0.63–1.07)	0.1485
Other	24 (2.4%)	0.70(0.39–1.23)	0.2182
Unknown	36 (3.7%)	0.95(0.60–1.52)	0.8598
**Sex**			
Female	577 (58.6%)	Reference	
Male	407 (41.4%)	1.23(1.04–1.45)	0.0108
**Stage**			
Stage I	74 (7.6%)	Reference	
Stage II	57 (5.8%)	0.79(0.51–1.21)	0.2862
Stage Ill	159 (16.2%)	0.79(0.56–1.12)	0.2031
Stage IV	693 (70.4%)	1.07(0.79–1.44)	0.6320
**Smoking**			
Current user	108 (11.0%)	Reference	
Former user	643 (65.3%)	1.03(0.81–1.32)	0.7705
Never used	233 (23.7%)	0.94(0.70–1.25)	0.6795
**Histology**			
LUAD	849 (86.3%)	Reference	
LUSC	92 (9.3%)	0.98 (0.72–1.33)	0.9066
Other	43 (4.4%)	1.86 (1.28–2.69)	0.0010
**Regimen**			
Carboplatin,Pemetrexed Disodium	288 (29.3%)	Reference	
Bevacizumab, Carboplatin, Pemetrexed Disodium	125 (12.7%)	0.72(0.56–0.92)	0.0105
Carboplatin,Gemcitabine Hydrochloride	31 (3.2%)	1.17(0.73–1.87)	0.5046
Carboplatin,Paclitaxel	77 (7.8%)	0.90(0.66–1.23)	0.5338
Docetaxel	80 (8.1%)	2.17(1.63–2.90)	< 0.0001
Docetaxel, Ramucirumab	67 (6.8%)	2.05(1.53–2.75)	< 0.0001
Cisplatin,Bevacizumab, Pemetrexed Disodium	42 (4.3%)	0.68(0.45–1.01)	0.0602
Cisplatin,Etoposide	23 (2.3%)	0.51(0.29–0.90)	0.0214
Cisplatin,Pemetrexed Disodium	72 (7.3%)	0.38(0.26–0.56)	< 0.0001
Cisplatin,Vinorelbine Tartrate	3 (0.3%)	0.30(0.04–2.20)	0.2421
Gemcitabine Hydrochloride	60 (6.1%)	1.99(1.45–2.73)	< 0.0001
Pemetrexed Disodium	66 (6.7%)	0.96(0.69–1.32)	0.8054
Vinorelbine Tartrate	50 (5.1%)	1.87(1.35–2.60)	0.0002

A noteworthy observation in our cohort was the significant impact of bevacizumab on PFS in carboplatin-based therapy, as expected, but not in cisplatin-based therapy (sFigure 1A, B). This observation does not aligns with earlier studies demonstrating that bevacizumab enhances chemotherapy outcomes in NSCLC [20, 21]. The discrepancy in our findings may be influenced by the significantly higher percentage of stage IV patients in the cisplatin + bevacizumab + pemetrexed-treated group (sTable 3). However, even though not statistically significant, stage IV patients treated with cisplatin + pemetrexed with bevacizumab still showed worse PFS compared to patients without bevacizumab treatment (sFigure 1C). This suggests that bevacizumab's efficacy in cisplatin-based therapy might warrant a larger-scale and well-controlled study.

Overall, the GENIE NSCLC BPC real-world data not only aligns with previous studies but also reveals nuanced differences, highlighting the complexity of treatment outcomes in this dynamic clinical landscape.

### Identification of prognostic mutations independent of driver genes in immune therapy

Key driver gene mutation status strongly influences patient responses to drugs, resulting in varied treatment approaches for patients with these mutations in clinical practice [22–24]. In our initial analysis, we aimed to include as many patients as possible. Consequently, we initially analyzed all patients before investigating the interaction of prognostic mutations with the driver mutations TP53, KRAS, and EGFR to determine whether the prognostic markers specifically apply to certain patient types.

To identify mutations associated with PFS, we conducted univariate Cox proportional hazards analyses, comparing PFS between patients with and without mutations in each gene. For patients receiving multiple ICI regimens, we analyzed the PFS of each regimen individually. Genes with mutation frequencies below 1% were excluded, as they often co-occurred, making it challenging to determine their significance as drivers or passengers.

**Fig. 2:**
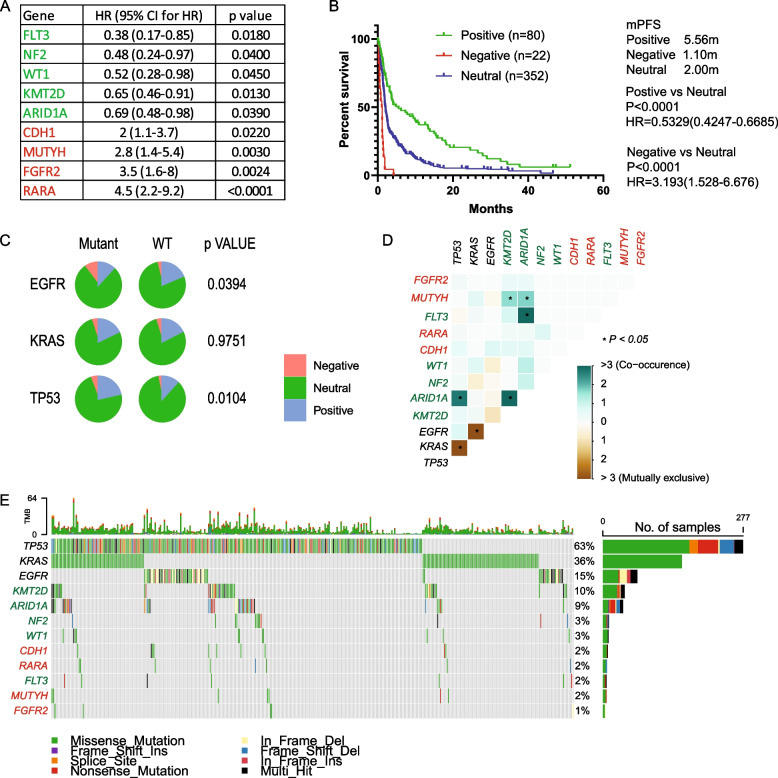
Mutation signature and prognostic impact in ICI therapy. **A**. Genes significantly associated with progression-free survival (PFS) identified by Cox proportional hazard analysis. **B**. Kaplan–Meier survival analysis depicting PFS for patients categorized into different groups based on mutational status, with log-rank tests assessing significance. **C**. Distribution of patients across Negative, Neutral, and Positive groups illustrated in a pie chart, stratified by EGFR, KRAS, and TP53 status. Statistical significance was evaluated by the Chi-square test. **D**. Co-occurrence analysis illustrating the interaction of the mutational signature with EGFR, KRAS, and TP53. Statistical significance was determined using Fisher’s exact test. **E**. Oncoplots visualizing the mutational signature alongside EGFR, KRAS, and TP53 mutations

We identified five positive prognostic mutations (FLT3, NF2, WT1, KMT2D, and ARID1A), significantly associated with improved PFS [hazard ratio (HR) < 1, *p* < 0.05, mutation rate > 1%] and four negative prognostic genes (CDH1, MUTYH, FGFR2, and RARA), significantly associated with worse PFS (HR > 1, *p* < 0.05, mutation rate > 1%) (Fig. 2A).

We next assigned the patients to distinct groups based on their mutation status. Patients exclusively harboring positive prognostic mutations were grouped as Positive, those with exclusively negative prognostic mutations were categorized as Negative, and individuals without prognostic mutations or with a combination were labeled as Neutral. Conducting Kaplan–Meier survival analysis on the three groups resulted in a median PFS of 5.56 months for the Positive group, 2.00 months for the Neutral group, and 1.10 months for the Negative group (Fig. 2B).

The interaction study of the signature with driver gene mutations revealed a significant association between EGFR and TP53 statuses, influencing the grouping of patients into the three prognostic groups (Fig. 2C). Next, we performed a co-occurrence analysis (Fig. 2D) and plotted the mutation details of driver and prognostic genes (Fig. 2E). We further assessed the performance of prognostic mutations in patients with different driver gene mutations. Our findings indicated that mutations in EGFR, KRAS, and TP53 did not significantly impact the prognostic power of the signature (sFigure 2A-C).

In conclusion, we identified five positive prognostic mutations (FLT3, NF2, WT1, KMT2D, and ARID1A) and four negative prognostic genes (CDH1, MUTYH, FGFR2, and RARA) in ICI-treated patients, and their predictive power was not associated with driver gene mutation status.

### Identification of prognostic mutations independent of driver genes in chemotherapy

In the chemotherapy setting, the most frequently administered drugs were carboplatin, cisplatin, pemetrexed, and docetaxel, constituting 88.8% of the chemotherapy regimens. Based on these four common drugs, we identified 521 carboplatin-based, 140 cisplatin-based, 593 pemetrexed-based, and 147 docetaxel-based treatment regimens. One regimens could be categorized into different groups; for example, the carboplatin + pemetrexed regimen was considered both carboplatin-based and pemetrexed-based. For patients with multiple chemotherapy regimens, we analyzed the PFS of each regimen individually.

**Fig. 3:**
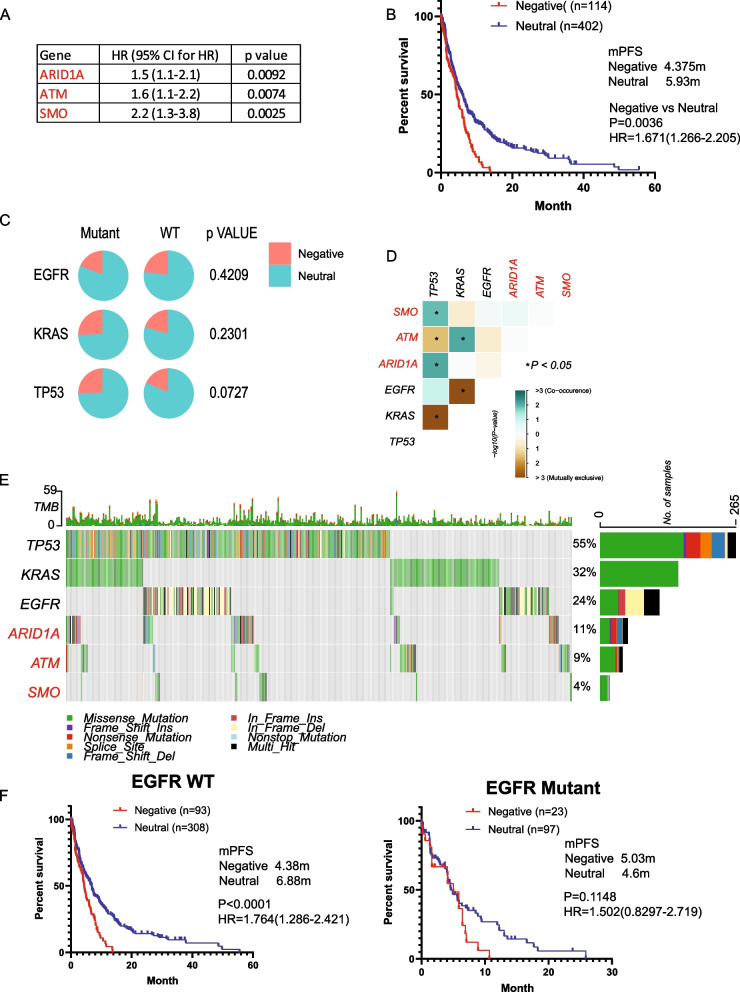
Mutation signature associated with prognosis and resistance in carboplatin-based chemotherapy. **A**. Genes significantly associated with progression-free survival (PFS) identified by Cox proportional hazard analysis. **B**. Kaplan–Meier survival analysis illustrating PFS for patients categorized into different groups based on mutational status, with significance assessed through a log-rank test. **C**. Distribution of patients across Negative and Neutral groups illustrated in a pie chart, stratified by EGFR, KRAS, and TP53 status, with statistical significance evaluated using the Chi-square test. **D**. Co-occurrence analysis depicting the interaction of the mutational signature with EGFR, KRAS, and TP53, with statistical significance determined using Fisher’s exact test. **E**. Oncoplots visualizing the mutational signature alongside EGFR, KRAS, and TP53 mutations. **F**. PFS of patients categorized into different groups based on mutational status in EGFR wild-type (WT) or EGFR mutant cases, assessed by the Kaplan–Meier method and log-rank test

Following a similar methodology, we identified three negative prognostic genes (ARID1A, ATM, and SMO) significantly associated with worse PFS (HR > 1, *p* < 0.05, mutation rate > 1%), with no positive prognostic genes identified (Fig. 3A). Kaplan–Meier survival analysis revealed a median PFS.

(mPFS) of 4.375 for the Negative group compared to 5.93 months for the Neutral group (Fig. 3B). A slightly higher percentage of Negative patients was observed in TP53 mutants compared to TP53 wild-type (WT) patients (Fig. 3C). Co-occurrence analysis demonstrated SMO and ARID1A co-occurred with TP53, and ATM co-occurred with KRAS but was mutually exclusive with TP53 (Fig. 3D, E). Kaplan–Meier survival analysis indicated similar efficacy of the prognostic mutation in both P53 or KRAS mutant and WT groups (sFigure 3A, B). However, the prognostic signature did not perform well in EGFR mutant patients (Fig. 3F).

**Fig. 4:**
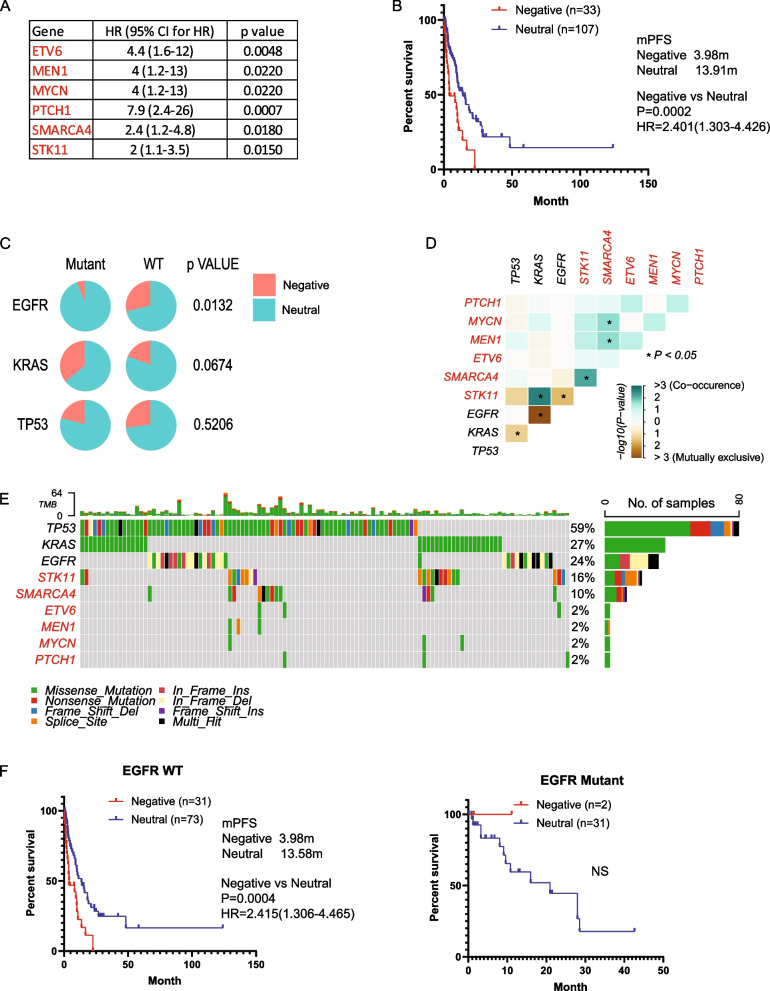
Mutation signature associated with prognosis and resistance in cisplatin-based chemotherapy. **A**. Genes significantly associated with progression-free survival (PFS) identified by Cox proportional hazard analysis. **B**. Kaplan–Meier survival analysis illustrating PFS for patients categorized into different groups based on mutational status, with significance assessed through the log-rank test. **C**. Distribution of patients across Negative and Neutral groups illustrated in a pie chart, stratified by EGFR, KRAS, and TP53 status, with statistical significance evaluated using the Chi-square test. **D**. Co-occurrence analysis depicting the interaction of the mutational signature with EGFR, KRAS, and TP53, with statistical significance determined using Fisher’s exact test. **E**. Oncoplots visualizing the mutational signature alongside EGFR, KRAS, and TP53 mutations. **F**. PFS of patients categorized into different groups based on mutational status in EGFR wild-type (WT) or EGFR mutant cases, assessed by the Kaplan–Meier method and log-rank test

In cisplatin-based regimens, we identified six negative prognostic genes (ETV6, MEN1, MYCN, PTCH1, SMARCA4, and STK11) significantly associated with worse PFS (HR > 1, *p* < 0.05, mutation rate > 1%), with no positive prognostic genes identified (Fig. 4A). Kaplan–Meier survival analysis showed a mPFS of 3.98 months for the Negative group compared to 13.91 months for the Neutral group (Fig. 4B). A lower percentage of patients was assigned to the Negative group in EGFR mutant cases compared to EGFR WT patients. Additionally, a significantly higher percentage of patients in the Negative group was found in KRAS mutant cases compared to KRAS WT patients (Fig. 4C). In co-occurrence analysis, we observed a strong mutual exclusion of the prognostic signature with EGFR (Fig. 4D, E). Kaplan–Meier survival analysis revealed that the prognostic mutation signature only worked with EGFR WT patients and not in EGFR mutant patients (Fig. 4F). KRAS and p53 status were not associated with the performance of the prognostic signature (sFigure 4A, B).

**Fig. 5:**
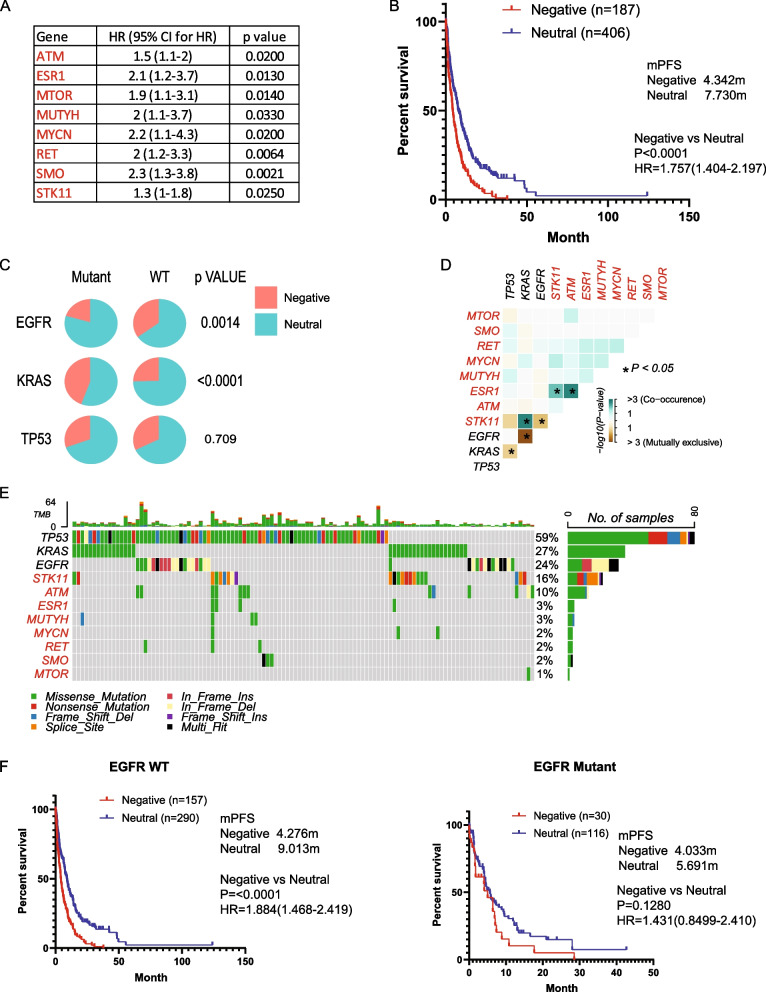
Mutation signature associated with prognosis and resistance in pemetrexed-based chemotherapy. **A**. Genes significantly associated with progression-free survival (PFS) identified by Cox proportional hazard analysis. **B**. Kaplan–Meier survival analysis illustrating PFS for patients categorized into different groups based on mutational status, with significance assessed through a log-rank test. **C**. Distribution of patients across Negative and Neutral groups illustrated in a pie chart, stratified by EGFR, KRAS, and TP53 status, with statistical significance evaluated using the Chi-square test. **D**. Co-occurrence analysis depicting the interaction of the mutational signature with EGFR, KRAS, and TP53, with statistical significance determined using Fisher’s exact test. **E**. Oncoplots visualizing the mutational signature alongside EGFR, KRAS, and TP53 mutations. **F**. PFS of patients categorized into different groups based on mutational status in EGFR wild-type (WT) or EGFR mutant cases, assessed by the Kaplan–Meier method and log-rank test

We analyzed patients treated with pemetrexed-based therapy, identifying eight negative prognostic mutations (ATM, ESR1, MTOR, MUTYH, MYCN, RET, SMO, and STK11) significantly associated with worse PFS (HR > 1, *p* < 0.05, mutation rate > 1%), with no positive prognostic genes identified (Fig. 5A). Several genes overlapped between the pemetrexed-based signature and those associated with carboplatin- and cisplatin-based therapies. Given that pemetrexed is often prescribed alongside carboplatin and cisplatin, constituting 78.9% of the pemetrexed-based regimen, there was suspicion that these genes might be specifically associated with the response to carboplatin or cisplatin, rather than pemetrexed. However, even in pemetrexed monotherapy-treated patients, the signature exhibited a powerful prognosis, although not statistically significant due to the low patient number (sFigure 5A), indicating a strong association with the response to pemetrexed.

Subsequently, Kaplan–Meier survival analysis revealed a mPFS of 4.34 months for the Negative group compared to 7.73 months for the Neutral group (Fig. 5B). Patient distribution in the Negative and Neutral groups showed a significant, strong association with EGFR and KRAS mutation status (Fig. 5C). Mutual exclusivity of the signature with EGFR resulted in a significantly lower percentage of patients placed in the Negative group among those with EGFR mutations (Fig. 5D, E). Additionally, the co-occurrence of the signature with KRAS led to a higher percentage of patients being assigned to the Negative group among those with KRAS mutations (Fig. 5D, E). Further investigation into the performance of the prognostic signature in patients with different driver gene mutations revealed better efficacy with EGFR WT patients compared to EGFR mutant patients (Fig. 5F). KRAS and p53 status were not associated with the performance of the prognostic signature (sFigure 5B, C).

**Fig. 6:**
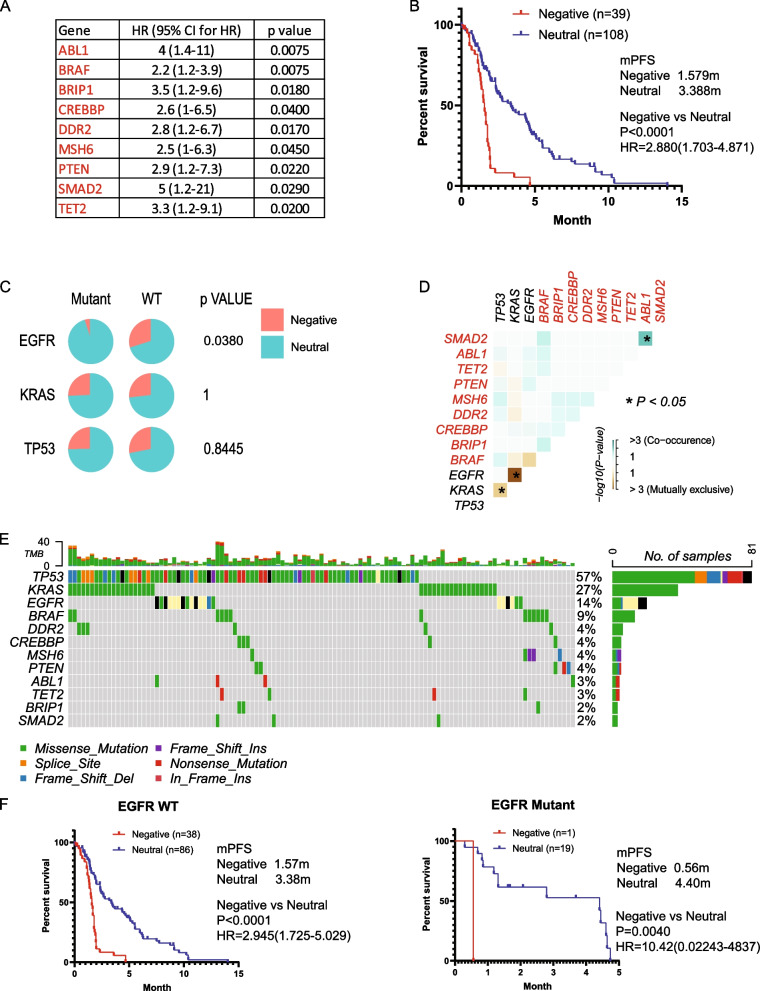
Mutation signature associated with prognosis and resistance in docetaxel-based chemotherapy. **A**. Genes significantly associated with progression-free survival (PFS) identified by Cox proportional hazard analysis. **B**. Kaplan–Meier survival analysis illustrating PFS of patients categorized into different groups based on mutational status, with significance assessed through a log-rank test. **C**. Distribution of patients across Negative and Neutral groups illustrated in a pie chart, stratified by EGFR, KRAS, and TP53 status, with statistical significance evaluated using the Chi-square test. **D**. Co-occurrence analysis depicting the interaction of the mutational signature with EGFR, KRAS, and TP53, with statistical significance determined using Fisher’s exact test. **E**. Oncoplots visualizing the mutational signature alongside EGFR, KRAS, and TP53 mutations. **F**. PFS of patients categorized into different groups based on mutational status in EGFR wild-type (WT) or EGFR mutant cases, assessed by the Kaplan–Meier method and log-rank test

In the analysis of patients treated with docetaxel-based therapy, we identified nine negative prognostic mutations (ABL1, BRAF, BRIP, CREBBP, DDR2, MSH6, PTEN, SMAD2, and TET2) significantly associated with worse PFS (HR > 1, *p* < 0.05, mutation rate > 1%), with no positive prognostic genes identified (Fig. 6A). The mPFS of the Negative and Neutral groups were 1.598 months and 3.39 months, respectively (Fig. 6B). Patient distribution was significantly associated with EGFR mutation status (Fig. 6C). We observed mutual exclusion of the BRAF mutation with the EGFR mutation, and BRAF co-occurred with almost all the other genes in the prognostic signature (Fig. 6D, E). This resulted in the underrepresentation of the Negative group in the EGFR mutant group. Consequently, our prognostic marker is specifically suitable for EGFR WT patients, as there are too few Negative patients to validate our result in EGFR mutant patients (Fig. 6F). KRAS and p53 status were not associated with the performance of the prognostic signature (sFigure 6A, B).

**Fig. 7:**
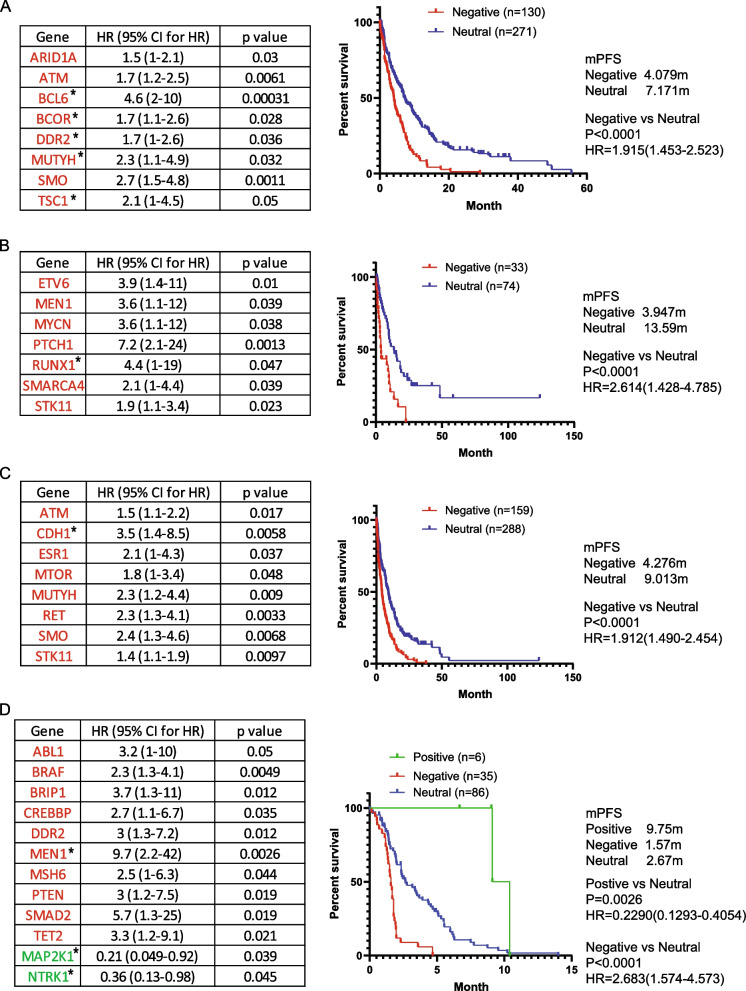
Mutation signature in EGFR wild-type (WT) patients during chemotherapy. **A**-**D**. Genes significantly associated with progression-free survival (PFS) during carboplatin-, cisplatin-, pemetrexed-, and docetaxel-based chemotherapy, respectively, were identified by Cox proportional hazard analysis. Kaplan–Meier survival analysis illustrating PFS for patients categorized into different groups based on mutational status, with significance assessed through a log-rank test. * New genes compared to signature for all patients

Given the significant association of the prognostic power of the signature with EGFR mutation status, we subsequently developed an EGFR WT-specific mutation signature for the chemotherapy regimens (Fig. 7A-D). In carboplatin-based therapy, five new genes (BCL6, BCOR, DDR2, MUTYH, and TSC1) were identified as negative prognostic markers, showing a significant difference compared to the previous signature. Conversely, in the other three groups, the signature exhibited only minor differences compared to previous signatures.

## Discussion

In recent years, NSCLC treatment has evolved with advances in targeted therapy and immunotherapy [4, 5]. The growing array of treatment options, characterized by lower toxicity, fewer side effects, and greater administration convenience, has significantly reduced the preference for chemotherapy among both patients and clinicians [4, 7]. Previously, when patients developed resistance to a specific chemotherapy, the typical approach involved selecting an alternative chemotherapy option, and repeating this process until resistance to all available chemotherapies emerged. However, contemporary patients who develop resistance to chemotherapy are increasingly inclined to choose ICIs or targeted therapy if applicable. Consequently, selecting the optimal chemotherapy is crucial for achieving the best outcome.

Our regimen-specific signature analysis can aid clinicians in selecting the most appropriate chemotherapy by analyzing patients’ mutations. For example, cisplatin is typically administered to patients with a good performance score, as studies indicate better PFS outcomes despite increased side effects [18, 25]. However, if a patient is predicted to have a negative prognosis for cisplatin-based treatment using the mutational signature, it is likely that they will not experience the extended PFS benefits associated with cisplatin. In such cases, alternative chemotherapy options should be considered. Several studies have demonstrated the predictive role of somatic mutations in determining responses to various therapies [7, 8, 26, 27]. However, these studies often contend with limitations such as small sample sizes, insufficient treatment information, or a narrow focus on individual genes. In contrast, our study leverages the expansive GENIE BPC cohort, representing the largest dataset to date, providing comprehensive clinicogenomic data for an in-depth analysis of somatic mutational signatures associated with drug responses in NSCLC patients. We systematically examined 158 somatic mutations and elucidated a prognostic signature linked to ICIs and the four primary types of chemotherapy widely employed in NSCLC treatment.

In the interaction analysis of prognostic mutations with the driver mutations TP53, KRAS, and EGFR, we observed that the EGFR mutation status significantly impacts the prognostic power of our signature in chemotherapy but not in immunotherapy. Surprisingly, TP53 mutation status did not show a significant impact on prognostic power, contrary to many previous studies that have emphasized the role of p53 mutations in treatment response. P53 mutations are generally thought to be related to improved immune checkpoint inhibitor (ICI) response due to increased mutation burden [23, 28]. However, recent studies have indicated that TP53 mutation serves as a negative prognostic marker in patients treated with ICIs [29, 30]. In the context of chemotherapy, the role of TP53 mutation as a prognostic marker remains controversial. Early studies suggested a better response to chemotherapy, especially cisplatin, in association with TP53 mutations [31, 32]. Conversely, recent studies, including our cohort, have shown that TP53 is not associated with chemotherapy response[33] [34]. This controversy may be attributed to the complexity of TP53 mutation heterogeneity [35]. Specific missense mutations of p53 result in the synthesis of a stable full-length protein that can act as a dominant negative form inhibiting wild-type TP53 expressed by the remaining allele or as a gain-of-function form resulting in a dominant oncogenic TP53. In contrast, less frequent nonsense mutations lead to a truncated inactive p53 protein, resulting in p53 deficiency. Additionally, some missense mutations have unknown effects. Studies have shown that TP53 missense mutations, but not nonsense mutations, are associated with better outcomes in ICI-treated patients, suggesting that the type of TP53 mutation should be carefully considered when using it as a biomarker [36]. Technological advancements have also contributed to the controversy. Early studies focused on hotspot mutations with known functions. However, with the high efficiency of next-generation sequencing (NGS), recent data on TP53 mutations include a large number of variants of uncertain significance (VUS), further complicating its role as a prognostic biomarker.

In our cohort, the overall TP53 status was not significantly associated with progression-free survival (PFS) for either treatment. However, we did observe an impact of TP53 status on prognostic group assignment. In ICI-treated patients, the percentage of patients assigned to the positive prognosis group was higher in the TP53 mutant group (21.4%) compared to the TP53 wild-type group (12.2%). Conversely, in the cisplatin-based treatment group, a higher percentage of patients was assigned to the negative prognosis group in the TP53 wild-type group (28.5%) compared to the TP53 mutant group (19.2%). These results suggest that TP53 mutations might be related to better outcomes in both ICI and cisplatin treatments but should be considered alongside other factors such as the mutation landscape of other genes and the type of TP53 mutation. The Food and Drug Administration’s (FDA) approval of ICIs in combination with chemotherapy in 2018 represented a significant milestone in NSCLC treatment, firmly establishing combination therapy as a major option in clinical practice [7, 37]. Unfortunately, a notable limitation of the GENIE BPC cohort is that most patient data were documented from 2014 to 2018, predating the FDA’s approval of this combination therapy. Consequently, there is an urgent need for continuous updates to patient data, particularly for those treated with combination therapy, to align with the evolving landscape of NSCLC treatments in clinical practice.

With the rapid growth of genomic profiling and the accumulation of data, an increasing number of clinicogenomic cohorts have become available. However, the GENIE BPC cohort remains the only publicly available large-scale cohort with curated, detailed treatment and response information. Consequently, validating our results in other cohorts is challenging. There is an urgent need for organizations worldwide to collaborate and build comprehensive data registries to accelerate clinicogenomic studies. Although we are unable to validate our signature because of lack of comparable cohort, many genes in our signature have been extensively reported and validated for their relevance to drug responses by individual studies. Notably, the KMT2D mutation has emerged as a positive prognostic marker for ICIs, demonstrating its capacity to sensitize tumors to ICIs, as supported by a CRISPR screening study [38, 39]. Furthermore, ARID1A mutation, widely acknowledged for its association with longer PFS following ICI treatment, has been linked to the promotion of mutability, resulting in an increased mutation burden [40–42]. In contrast to findings in immunotherapy, where several studies suggest that ARID1A mutation is associated with chemotherapy resistance [43, 44], our study presents a novel perspective. Specifically, our findings reveal that ARID1A mutation is exclusively linked to an adverse outcome in carboplatin-based therapy but not in cisplatin-, pemetrexed-, or docetaxel-based chemotherapy. Moreover, STK11, previously reported to be associated with chemoresistance [45], exhibits a distinctive pattern in our study. Our results indicate that STK11 alone is specifically linked to worse PFS in cisplatin- or pemetrexed-based therapy but not in carboplatin- or docetaxel-based therapy.

Alterations in DNA repair processes play a crucial role in mediating chemotherapy resistance [46, 47]. The majority of previous studies have demonstrated that chemoresistance is linked to increased DNA damage repair, and inhibitors of DNA repair pathways have the potential to sensitize cancer cells to chemotherapy [48]. However, concerns have been raised regarding the use of DNA repair inhibitors, as they may increase mutagenic lesions and contribute to tumor development [48]. Our study identified multiple DNA repair gene mutations, including ATM, BRIP1, MUTYH, and MSH6, associated with poor PFS in chemotherapy. This finding suggests that the loss of specific DNA repair functions may be associated with a worse clinical outcome, highlighting the potential multifaceted impact of DNA repair inhibitors in patients.

## Conclusions

Within the domain of NSCLC, our study initially identified drug-specific mutation prognostic markers for ICI therapy and various chemotherapy regimens, including carboplatin, cisplatin, pemetrexed, and docetaxel. Subsequent mutation interaction analyses unveiled intricate relationships, particularly with EGFR mutations in chemotherapy. Notably, EGFR mutations either exhibited mutual exclusivity with the prognostic signature, resulting in the underrepresentation of the Negative group in EGFR mutant patients, or significantly influenced the prognostic power of the signature. Consequently, we developed a targeted EGFR WT-specific prognostic marker for chemotherapy. Our prognostic signature consistently aligns with prior studies and holds promise in facilitating clinical decision-making, especially in scenarios where multiple treatment options are available for NSCLC patients.

### Supplementary Information

Supplementary Material 1.

Supplementary Material 2: Supplementary Figure 1. Progression-free survival (PFS) of patients treated with a platinum-based regimen with or without bevacizumab.A-C. Kaplan–Meier survival analysis illustrating PFS for patients treated with carboplatin + pemetrexed, cisplatin + pemetrexed, and stage V patients treated with cisplatin + pemetrexed, respectively, with or without bevacizumab. Significance was assessed using the log-rank test. Supplementary Figure 2. Prognostic impact of the mutation signature for immune checkpoint inhibitor (ICI) therapy in EGFR, KRAS, or TP53 mutant or wild-type (WT) patients. Progression-free survival (PFS) of patients categorized into Positive, Negative, or Neutral groups based on the mutational signature status of ICI therapy in (A) EGFR, (B) KRAS, and (C) TP53 WT or mutant cases, assessed using the Kaplan–Meier method with log-rank test. Supplementary Figure 3. Prognostic impact of the mutation signature in carboplatin-based chemotherapy in KRAS or TP53 mutant or wild-type (WT) patients. Progression-free survival (PFS) of patients categorized into Negative or Neutral groups based on the mutational signature status of carboplatin-based chemotherapy in (A) KRAS and (B) TP53 WT or mutant cases, assessed using the Kaplan–Meier method with log-rank test. Supplementary Figure 4. Prognostic impact of the mutation signature in cisplatin-based chemotherapy in KRAS or TP53 mutant or wild-type (WT) patients. Progression-free survival (PFS) of patients categorized into Negative or Neutral groups based on the mutational signature status of cisplatin-based chemotherapy in (A) KRAS and (B) TP53 WT or mutant cases, assessed using the Kaplan–Meier method with log-rank test. Supplementary Figure 5. Prognostic impact of the mutation signature in pemetrexed-based chemotherapy. A. Progression-free survival (PFS) of patients treated with pemetrexed monotherapy, categorized into Negative or Neutral groups based on the mutational signature status, assessed using the Kaplan–Meier method with a log-rank test. B-C. PFS of patients treated with pemetrexed-based chemotherapy, categorized into Negative or Neutral groups based on the mutational signature status in (B) KRAS and (C) TP53 WT or mutant cases, assessed using the Kaplan–Meier method with log-rank test. Supplementary Figure 6. Prognostic impact of the mutation signature in docetaxel-based chemotherapy. Progression-free survival (PFS) of patients treated with docetaxel-based chemotherapy, categorized into Negative or Neutral groups based on the mutational signature status of (A) KRAS and (B) TP53 wild-type (WT) or mutant cases, assessed using the Kaplan–Meier method with a log-rank test.

## Data Availability

Clinical data (GENIE BPC NSCLC v2.0-public) and genomic data (Release 13.0-public) are available from cBioPortal (https://genie.cbioportal.org) and Sage Bionetworks (https://sagebionetworks.org/community/aacr-project-genie), respectively.
