# Supplementary material for: Insights into treatment-specific prognostic somatic mutations in NSCLC from the AACR NSCLC GENIE BPC cohort analysis
Source: BMC Pulm Med. 2024 Jul 2;24:309. doi: 10.1186/s12890-024-03124-4 (PMC11218090; doi:10.1186/s12890-024-03124-4)
Supplement: Supplementary file 2 — Supplementary Material 2: Supplementary Figure 1. Progression-free survival (PFS) of patients treated with a platinum-based regimen with or without bevacizumab.A-C. Kaplan–Meier survival analysis illustrating PFS for patients treated with carboplatin + pemetrexed, cisplatin + pemetrexed, and stage V patients treated with cisplatin + pemetrexed, respectively, with or without bevacizumab. Significance was assessed using the log-rank test. Supplementary Figure 2. Prognostic impact of the mutation signature for immune checkpoint inhibitor (ICI) therapy in EGFR, KRAS, or TP53 mutant or wild-type (WT) patients. Progression-free survival (PFS) of patients categorized into Positive, Negative, or Neutral groups based on the mutational signature status of ICI therapy in (A) EGFR, (B) KRAS, and (C) TP53 WT or mutant cases, assessed using the Kaplan–Meier method with log-rank test. Supplementary Figure 3. Prognostic impact of the mutation signature in carboplatin-based chemotherapy in KRAS or TP53 mutant or wild-type (WT) patients. Progression-free survival (PFS) of patients categorized into Negative or Neutral groups based on the mutational signature status of carboplatin-based chemotherapy in (A) KRAS and (B) TP53 WT or mutant cases, assessed using the Kaplan–Meier method with log-rank test. Supplementary Figure 4. Prognostic impact of the mutation signature in cisplatin-based chemotherapy in KRAS or TP53 mutant or wild-type (WT) patients. Progression-free survival (PFS) of patients categorized into Negative or Neutral groups based on the mutational signature status of cisplatin-based chemotherapy in (A) KRAS and (B) TP53 WT or mutant cases, assessed using the Kaplan–Meier method with log-rank test. Supplementary Figure 5. Prognostic impact of the mutation signature in pemetrexed-based chemotherapy. A. Progression-free survival (PFS) of patients treated with pemetrexed monotherapy, categorized into Negative or Neutral groups based on the mutational signature status, assessed [file 12890_2024_3124_MOESM2_ESM.pdf]

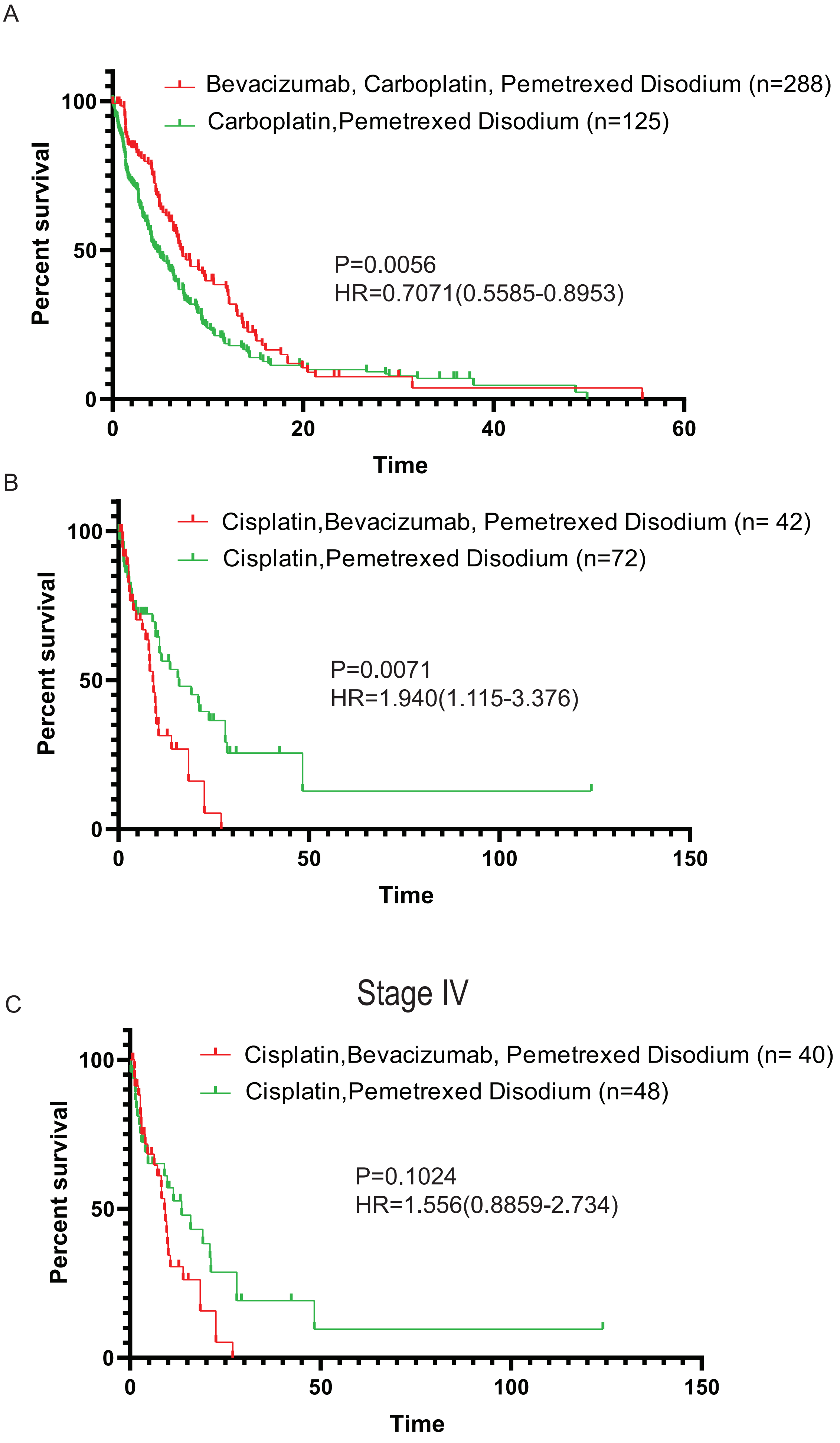

A
Bevacizumab, Carboplatin, Pemetrexed Disodium (n=288)
Carboplatin,Pemetrexed Disodium (n=125)
P=0.0056
HR=0.7071(0.5585-0.8953)
Percent survival
0
50
100
0
20
40
60
Time
B
Cisplatin,Bevacizumab, Pemetrexed Disodium (n= 42)
Cisplatin,Pemetrexed Disodium (n=72)
P=0.0071
HR=1.940(1.115-3.376)
Percent survival
0
50
100
0
50
100
150
Time
C
Stage IV
Cisplatin,Bevacizumab, Pemetrexed Disodium (n= 40)
Cisplatin,Pemetrexed Disodium (n=48)
P=0.1024
HR=1.556(0.8859-2.734)
Percent survival
0
50
100
0
50
100
150
Time

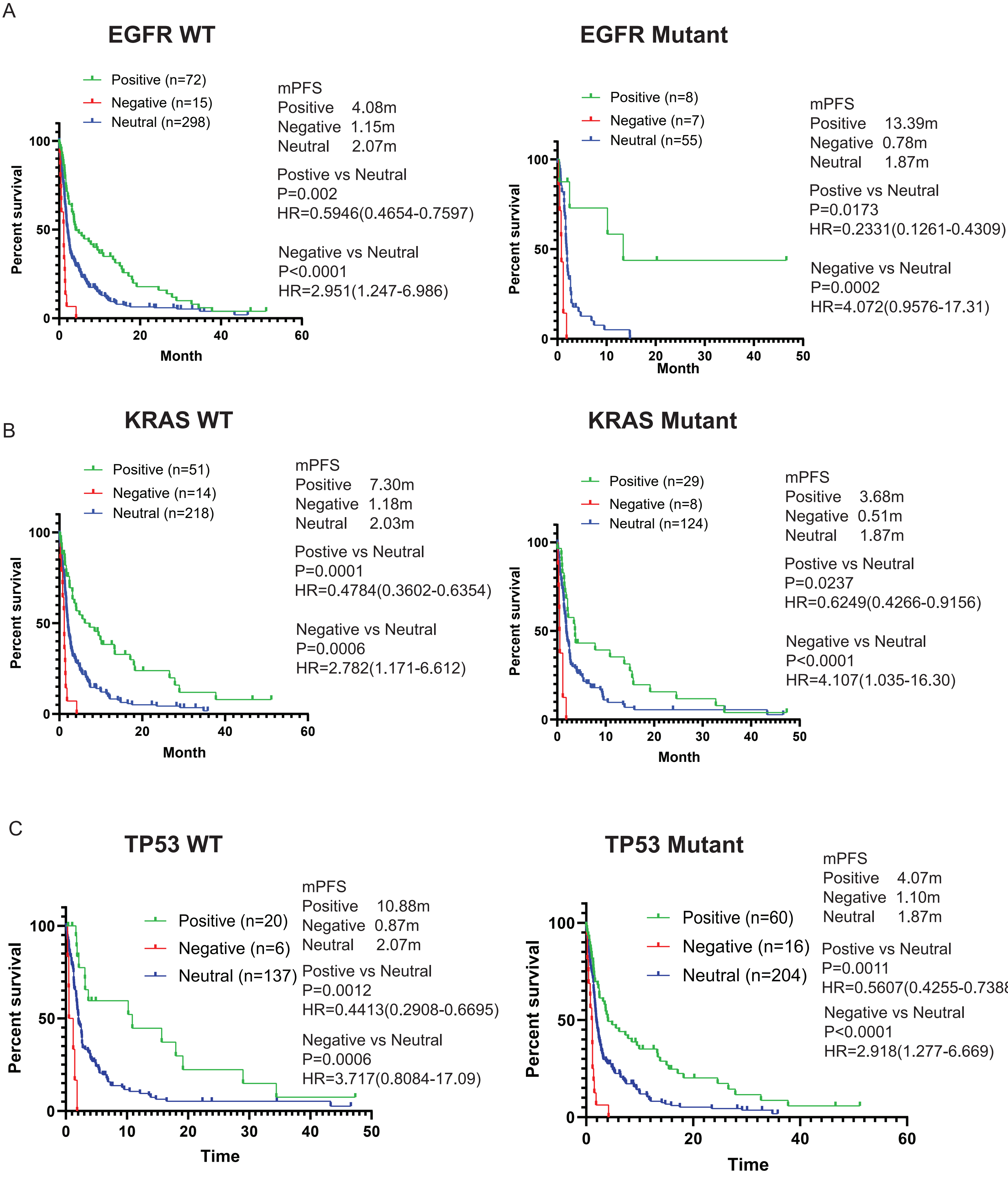

A
EGFR WT
Positive (n=72)
Negative (n=15)
Neutral (n=298)
mPFS
Positive 4.08m
Negative 1.15m
Neutral 2.07m
Postive vs Neutral
P=0.002
HR=0.5946(0.4654-0.7597)
Negative vs Neutral
P<0.0001
HR=2.951(1.247-6.986)
Percent survival
100
50
0
0
20
40
60
Month
EGFR Mutant
Positive (n=8)
Negative (n=7)
Neutral (n=55)
mPFS
Positive 13.39m
Negative 0.78m
Neutral 1.87m
Postive vs Neutral
P=0.0173
HR=0.2331(0.1261-0.4309)
Negative vs Neutral
P=0.0002
HR=4.072(0.9576-17.31)
Percent survival
100
50
0
0
10
20
30
40
50
Month
B
KRAS WT
Positive (n=51)
Negative (n=14)
Neutral (n=218)
mPFS
Positive 7.30m
Negative 1.18m
Neutral 2.03m
Postive vs Neutral
P=0.0001
HR=0.4784(0.3602-0.6354)
Negative vs Neutral
P=0.0006
HR=2.782(1.171-6.612)
Percent survival
100
50
0
0
20
40
60
Month
KRAS Mutant
Positive (n=29)
Negative (n=8)
Neutral (n=124)
mPFS
Positive 3.68m
Negative 0.51m
Neutral 1.87m
Postive vs Neutral
P=0.0237
HR=0.6249(0.4266-0.9156)
Negative vs Neutral
P<0.0001
HR=4.107(1.035-16.30)
Percent survival
100
50
0
0
10
20
30
40
50
Month
C
TP53 WT
Positive (n=20)
Negative (n=6)
Neutral (n=137)
mPFS
Positive 10.88m
Negative 0.87m
Neutral 2.07m
Postive vs Neutral
P=0.0012
HR=0.4413(0.2908-0.6695)
Negative vs Neutral
P=0.0006
HR=3.717(0.8084-17.09)
Percent survival
100
50
0
0
10
20
30
40
50
Time
TP53 Mutant
mPFS
Positive 4.07m
Negative 1.10m
Neutral 1.87m
Positive (n=60)
Negative (n=16)
Neutral (n=204)
Postive vs Neutral
P=0.0011
HR=0.5607(0.4255-0.7388
Negative vs Neutral
P<0.0001
HR=2.918(1.277-6.669)
Percent survival
100
50
0
0
20
40
60
Time

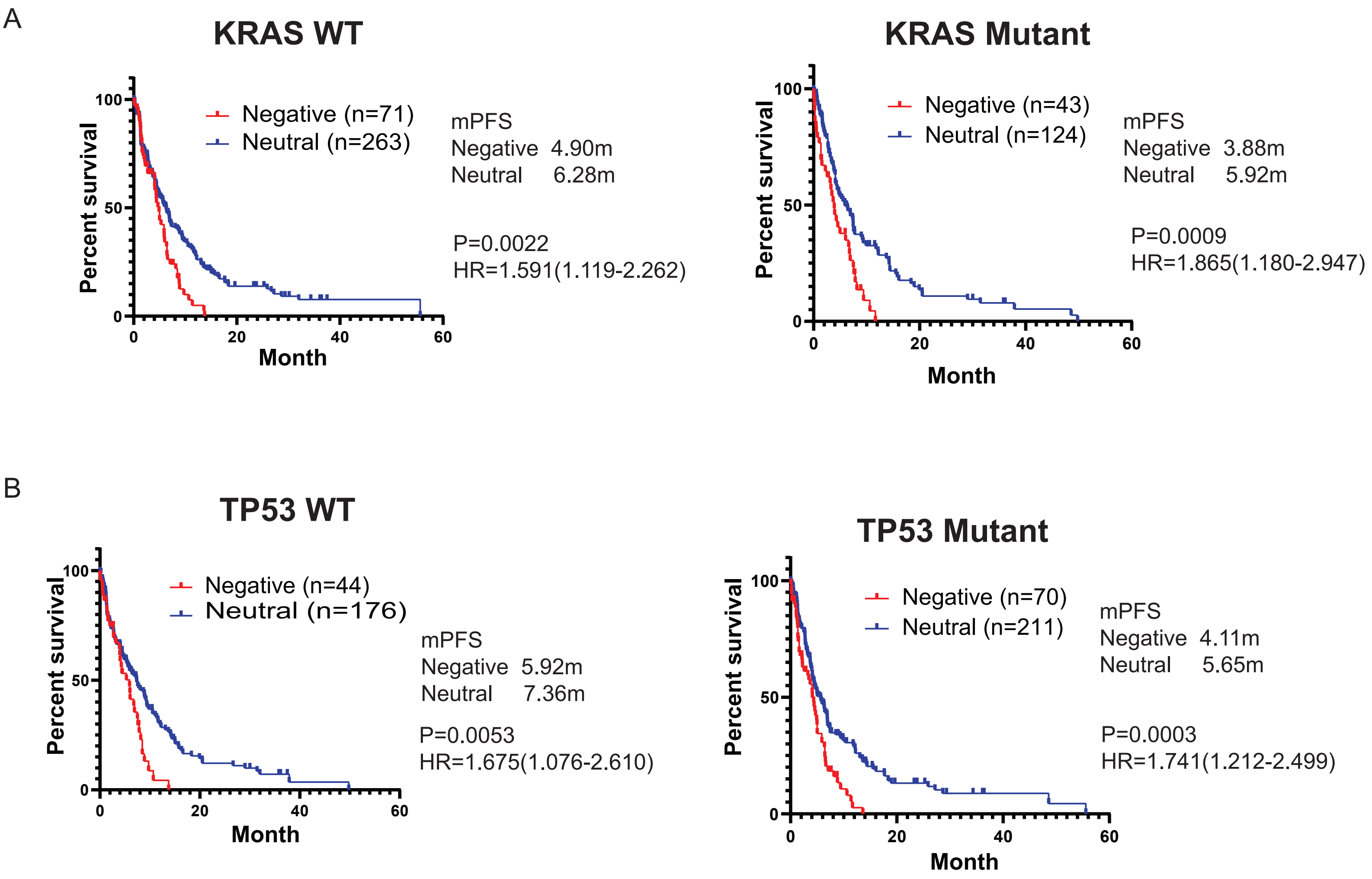
A
KRAS WT
Negative (n=71)
Neutral (n=263)
mPFS
Negative 4.90m
Neutral 6.28m
P=0.0022
HR=1.591(1.119-2.262)
Percent survival
Month
KRAS Mutant
Negative (n=43)
Neutral (n=124)
mPFS
Negative 3.88m
Neutral 5.92m
P=0.0009
HR=1.865(1.180-2.947)
Percent survival
Month
B
TP53 WT
Negative (n=44)
Neutral (n=176)
mPFS
Negative 5.92m
Neutral 7.36m
P=0.0053
HR=1.675(1.076-2.610)
Percent survival
Month
TP53 Mutant
Negative (n=70)
Neutral (n=211)
mPFS
Negative 4.11m
Neutral 5.65m
P=0.0003
HR=1.741(1.212-2.499)
Percent survival
Month

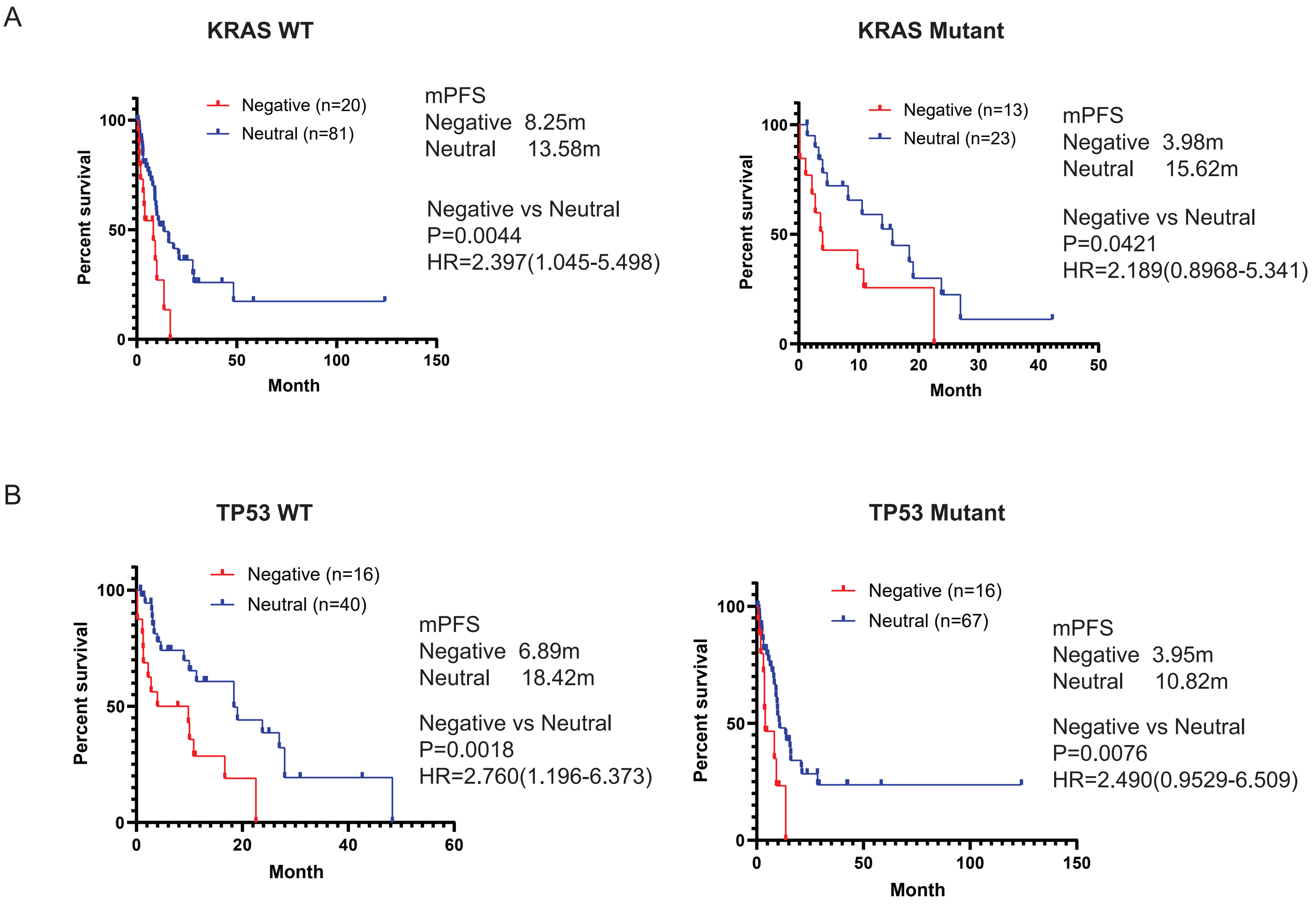
A
KRAS WT
Negative (n=20)
Neutral (n=81)
mPFS
Negative 8.25m
Neutral 13.58m
Negative vs Neutral
P=0.0044
HR=2.397(1.045-5.498)
Percent survival
Month
KRAS Mutant
Negative (n=13)
Neutral (n=23)
mPFS
Negative 3.98m
Neutral 15.62m
Negative vs Neutral
P=0.0421
HR=2.189(0.8968-5.341)
Percent survival
Month
B
TP53 WT
Negative (n=16)
Neutral (n=40)
mPFS
Negative 6.89m
Neutral 18.42m
Negative vs Neutral
P=0.0018
HR=2.760(1.196-6.373)
Percent survival
Month
TP53 Mutant
Negative (n=16)
Neutral (n=67)
mPFS
Negative 3.95m
Neutral 10.82m
Negative vs Neutral
P=0.0076
HR=2.490(0.9529-6.509)
Percent survival
Month

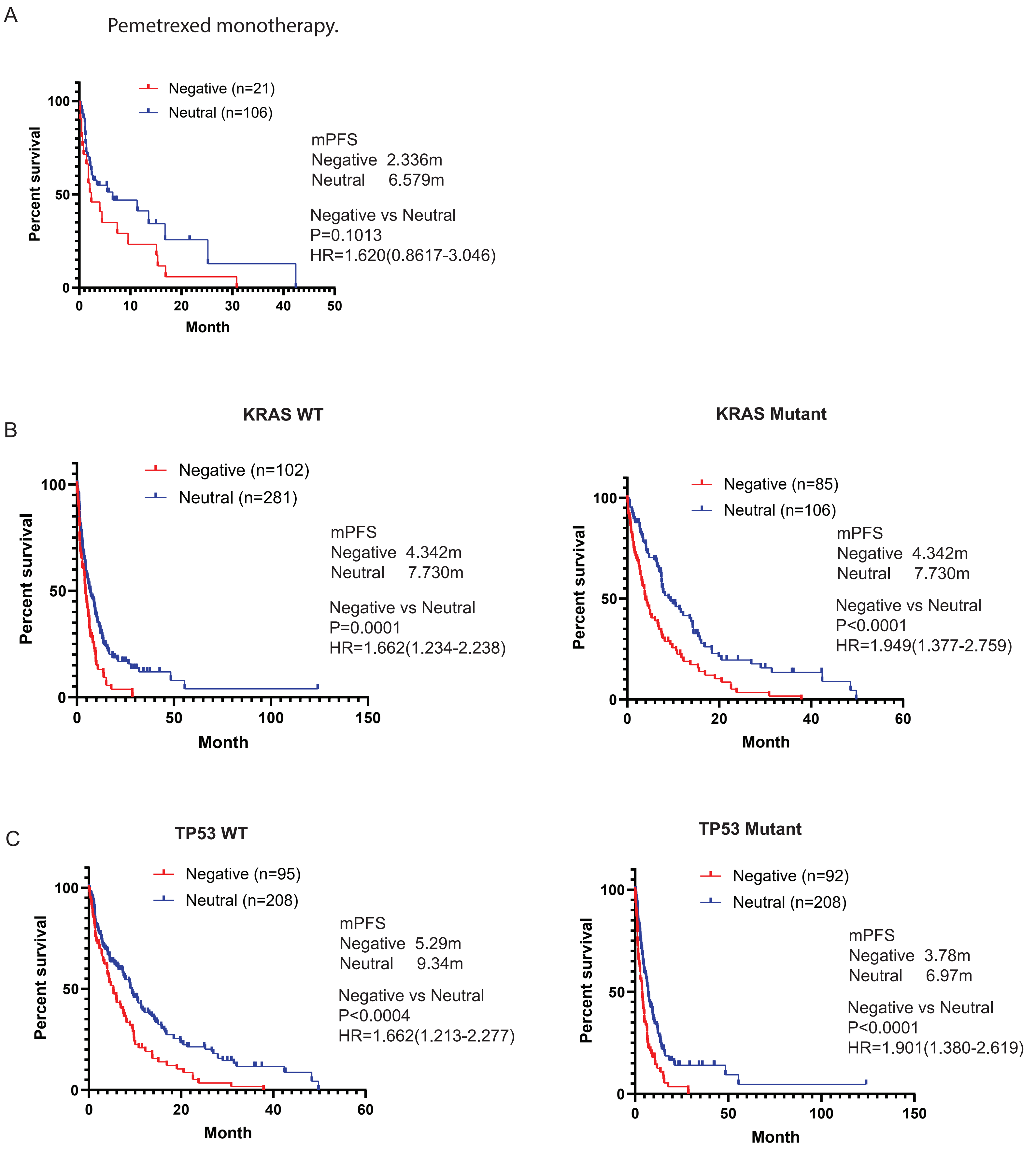

A
Pemetrexed monotherapy.
Negative (n=21)
Neutral (n=106)
mPFS
Negative 2.336m
Neutral 6.579m
Negative vs Neutral
P=0.1013
HR=1.620(0.8617-3.046)
Percent survival
Month
B
KRAS WT
Negative (n=102)
Neutral (n=281)
mPFS
Negative 4.342m
Neutral 7.730m
Negative vs Neutral
P=0.0001
HR=1.662(1.234-2.238)
Percent survival
Month
KRAS Mutant
Negative (n=85)
Neutral (n=106)
mPFS
Negative 4.342m
Neutral 7.730m
Negative vs Neutral
P<0.0001
HR=1.949(1.377-2.759)
Percent survival
Month
C
TP53 WT
Negative (n=95)
Neutral (n=208)
mPFS
Negative 5.29m
Neutral 9.34m
Negative vs Neutral
P<0.0004
HR=1.662(1.213-2.277)
Percent survival
Month
TP53 Mutant
Negative (n=92)
Neutral (n=208)
mPFS
Negative 3.78m
Neutral 6.97m
Negative vs Neutral
P<0.0001
HR=1.901(1.380-2.619)
Percent survival
Month

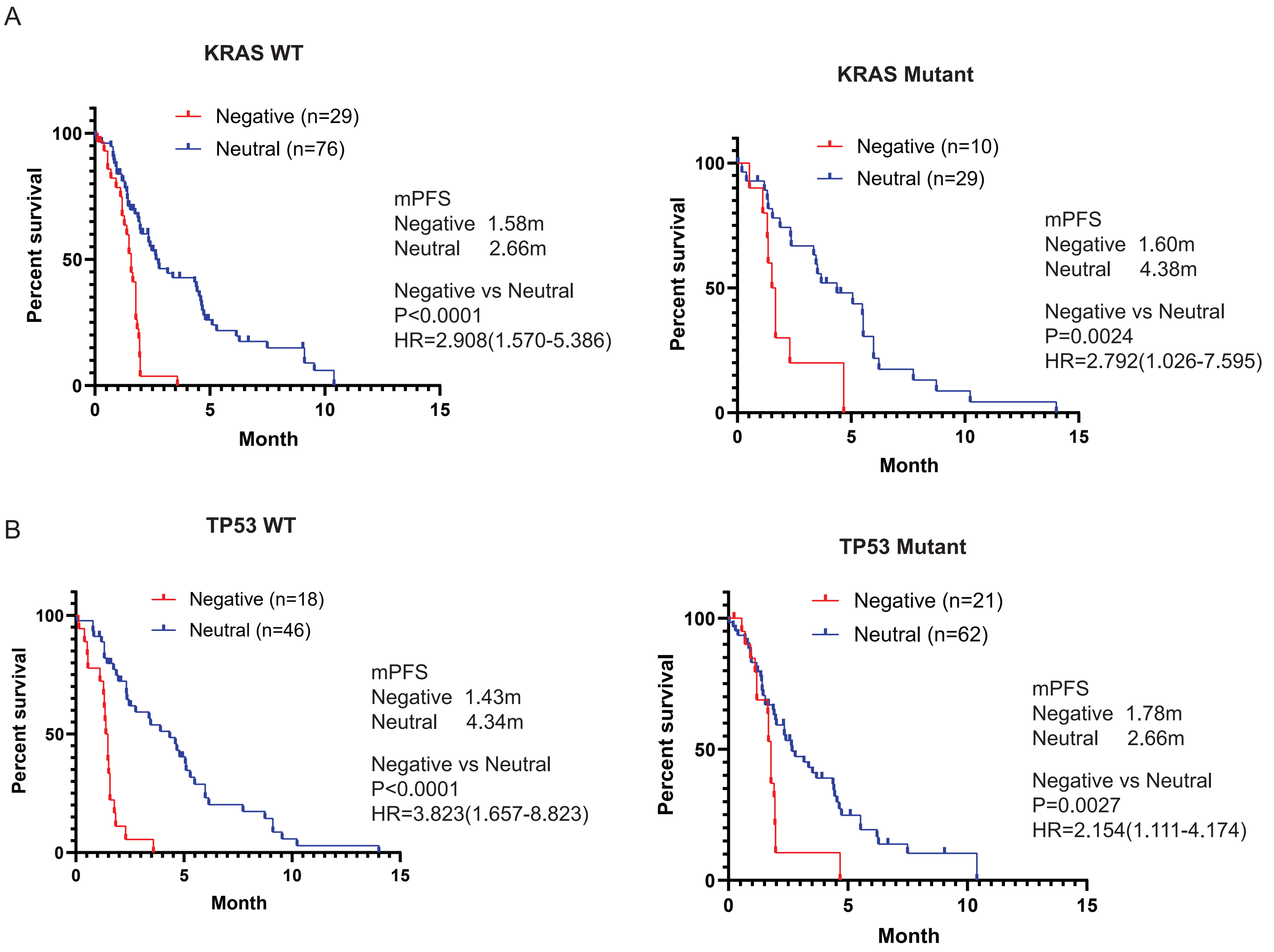
A
KRAS WT
Negative (n=29)
Neutral (n=76)
Percent survival
100
50
0
0
5
10
15
Month
mPFS
Negative 1.58m
Neutral 2.66m
Negative vs Neutral
P<0.0001
HR=2.908(1.570-5.386)
KRAS Mutant
Negative (n=10)
Neutral (n=29)
mPFS
Negative 1.60m
Neutral 4.38m
Negative vs Neutral
P=0.0024
HR=2.792(1.026-7.595)
B
TP53 WT
Negative (n=18)
Neutral (n=46)
mPFS
Negative 1.43m
Neutral 4.34m
Negative vs Neutral
P<0.0001
HR=3.823(1.657-8.823)
TP53 Mutant
Negative (n=21)
Neutral (n=62)
mPFS
Negative 1.78m
Neutral 2.66m
Negative vs Neutral
P=0.0027
HR=2.154(1.111-4.174)
